# Neuroinflammation in a Mouse Model of Alzheimer’s Disease versus Auditory Dysfunction: Machine Learning Interpretation and Analysis

**DOI:** 10.21203/rs.3.rs-3370200/v1

**Published:** 2023-09-27

**Authors:** Daxiang Na, Yidan Yang, Li Xie, Dorota Piekna-Przybylska, Dominic Bunn, Maleelo Shamambo, Patricia White

**Affiliations:** University of Rochester Medical Center; Rochester Institute of Technology; University of Rochester Medical Center; University of Rochester Medical Center; University of Rochester Medical Center; University of Rochester Medical Center; University of Rochester Medical Center

**Keywords:** Alzheimer’s Disease, mouse amyloidosis models, machine learning, central auditory hyperactivity, microglial activation, astrocyte activation, neuroinflammation, inferior colliculus, auditory brainstem response

## Abstract

**Background:**

Auditory dysfunction, including central auditory hyperactivity, hearing loss and hearing in noise deficits, has been reported in 5xFAD Alzheimer’s disease (AD) mice, suggesting a causal relationship between amyloidosis and auditory dysfunction. Central auditory hyperactivity correlated in time with small amounts of plaque deposition in the inferior colliculus and medial geniculate body, which are the auditory midbrain and thalamus, respectively. Neuroinflammation has been associated with excitation to inhibition imbalance in the central nervous system, and therefore has been proposed as a link between central auditory hyperactivity and AD in our previous report. However, neuroinflammation in the auditory pathway has not been investigated in mouse amyloidosis models.

**Methods:**

Machine learning was used to classify the previously obtained auditory brainstem responses (ABRs) from 5xFAD mice and their wild type (WT) littermates. Neuroinflammation was assessed in six auditory-related regions of the cortex, thalamus, and brainstem. Cochlear pathology was assessed in cryosection and whole mount. Behavioral changes were assessed with fear conditioning, open field testing and novel objection recognition.

**Results:**

Reliable machine learning classification of 5xFAD and WT littermate ABRs were achieved for 6M and 12M, but not 3M. The top features for accurate classification at 6 months of age were characteristics of Waves IV and V. Microglial and astrocytic activation were pronounced in 5xFAD inferior colliculus and medial geniculate body at 6 months, two neural centers that are thought to contribute to these waves. Lower regions of the brainstem were unaffected, and cortical auditory centers also displayed inflammation beginning at 6 months. No losses were seen in numbers of spiral ganglion neurons (SGNs), auditory synapses, or efferent synapses in the cochlea. 5xFAD mice had reduced responses to tones in fear conditioning compared to WT littermates beginning at 6 months.

**Conclusions:**

Serial use of ABR in early AD patients represents a promising approach for early and inexpensive detection of neuroinflammation in higher auditory brainstem processing centers. As changes in auditory processing are strongly linked to AD progression, central auditory hyperactivity may serve as a biomarker for AD progression and/or stratify AD patients into distinct populations.

## Background

Previous studies in humans suggest that AD and auditory dysfunction are not only correlated at cortical level but also subcortical level. The hallmarks of AD, including amyloid-b plaques and neurofibrillary tangles, were found to be frequently present in the upper auditory brainstem, namely the medial geniculate body (MGB) and inferior colliculus (IC), in AD patients (Ohm and Braak, 1989; Sinha et al., 1993). A recent study reported auditory brainstem hyperactivity in patients diagnosed with mild cognitive impairment (MCI), a precursor for AD (Bidelman et al., 2017). The brainstem activity in this study was measured with frequency-following response (FFR), which is thought to reflect a neurophonic potential generated in the IC (Sohmer et al., 1977; Bidelman and Alain, 2015; Coffey et al., 2019). Together, the physiological and histological evidence suggests the correlation between AD and brainstem auditory hyperactivity in humans.

We previously reported that a mouse model of amyloidosis, 5xFAD, exhibited central hyperactivity in the auditory brainstem (Na et al., 2023), suggesting the application of this model for investigating the correlation between auditory dysfunction and AD. The brainstem hyperactivity became significant in 5xFAD mice at 6M, prior to ABR Wave I amplitude reduction and hearing loss (Na et al., 2023), which suggests the central origin of this hyperactivity. Histological examination has revealed amyloid-β plaque in ABR generators, which provides the direct evidence for the correlation between the amyloid-β plaque and central auditory hyperactivity in the brainstem(Na et al., 2023). In addition to this correlation, the hearing loss and hearing in noise deficit, which are similar to symptoms reported in AD patients (Swords et al., 2018), also manifested in 5xFAD mice (Na et al., 2023).

Neuroinflammation has been proposed as the link between AD and central auditory hyperactivity (Na et al., 2023). However, the status of inflammatory response, including the microglia and astrocyte activity, in auditory brainstem regions remains undetermined. To further examine the correlation between amyloid deposition and central auditory hyperactivity, how neuroinflammation spatially and temporally correlates with brainstem activity changes still needs to be determined. Beyond central auditory hyperactivity, the origin of hearing dysfunction, including hearing loss and hearing in noise deficit, also remains unclear. Those dysfunctions could originate from damage in cochlear structures, including SGNs, afferent and efferent terminals. In addition, cochlear synaptic loss can also possibly contribute to central auditory hyperactivity (Möhrle et al., 2016). Examination of these cochlear structures is needed to further understand the correlation between AD and those hearing dysfunction.

The present study aims to further examine the correlation between AD and auditory dysfunction in 5xFAD mice. To achieve this goal, we first evaluated whether AD can be identified by a combination of ABR and machine learning approach in 5xFAD mice. Based on the successful AD identification, we were able to further analyze the brainstem activity changes in 5xFAD mice via the interpretability of the machine learning algorithm. We then examined the spatial and temporal correlation between neuroinflammation and brainstem activity changes in 5xFAD mice. To characterize the impact of peripheral changes to the central auditory hyperactivity, hearing loss and hearing in noise in 5xFAD mice, we further examined the SGN, afferent and efferent terminal loss in the cochleae of 5xFAD mice at 12M, which is the age when all of those auditory dysfunctions manifested. To examine the temporal correlation between central auditory hyperactivity and cognitive decline in 5xFAD mice, a battery of behavioral assays, including open field testing, novel object recognition and fear conditioning, was performed on 5xFAD mice aged between 3M to 12M.

## Methods

### Mice

Mice used in this study were previously described (Na et al., 2023).Briefly, 5xFAD (Oakley et al., 2006) and APP/PS1 (Jankowsky et al., 2004) mouse lines with C57BL/6J congenic background were obtained from the Jackson Laboratory (Bar Harbor, ME, USA; stock no. 34848-JAX and 34832-JAX) and bred to wild type (WT) CBA/CaJ mice. Only F1 heterozygote 5xFAD males, heterozygote APP/PS1 males, and their WT male littermates were used in this study. The genotyping of tail samples was performed by Transnetyx Automated PCR Genotyping Services (Transnetyx, Inc. Cordova, TN, USA). The mouse breeding and housing routine were the same as described (Na et al., 2023). 5xFAD and APP/PS1 mice are collectively referred to as AD mice when not specified. All animal protocols were approved in advance by the University Committee for Animal Research (UCAR) at the University of Rochester Medical Center.

### Auditory Brainstem Response (ABR)

The ABRs were recorded as described (Na et al., 2023). Click-evoked ABRs were recorded and analyzed in this chapter. The ABRs consisted of 50-microsecond (ms) stimuli. Stimuli amplitudes decreased in 5 decibel (dB) steps from 75 dB sound pressure level to 5 dB. The averages of 512 sweeps were recorded for each amplitude.

ABR peaks and troughs were registered by a reviewer blinded to the experimental design and mouse genotypes as described (Na et al., 2023). Wave latency was defined as the difference between auditory stimuli onset (0 ms) to the time of the peak apex (ms). Wave amplitude was defined as the difference between the peak apex and the following trough (μV). All five waves (I, II, III, IV and V) were extracted in this study.

### Histology of the brain

Histological analysis was performed on brains of 5xFAD mice aged 3 months (3M), 6M, and 12M; APP/PS1 mice aged 13M, and WT littermates aged 12M (n = 6 for each age and genotype). Brains were dissected and sectioned as described (Na et al., 2023).

Sections containing the brain nuclei of interest were selected by comparison with the Mouse Brain Atlas (Franklin and Paxinos, 2019). Regions of the auditory cortex (AC), the medial geniculate body (MGB), the inferior colliculus (IC), the medial nucleus of the trapezoid body (MNTB), the superior olivary complex (SOC), and the cochlear nucleus (CN) were analyzed for potential correlates to auditory processing disorder, as described (Na et al., 2023). The following primary antibodies were used in this study: rabbit anti-Iba1 (1:2000, Wako - Sigma-Aldrich, St. Louis, MO, USA), rabbit anti-GFAP (1:1000, Novus Biologicals, Centennial, CO, USA), and rat anti-CD68 (1:500, Bio-Rad, Hercules, CA, USA). Floating sections were incubated in primary antibodies diluted in 0.5%Trion X-100/PBS (PBST) with 5% donkey serum overnight at 4°C. They were washed three times for 30 minutes in PBST with gentle rocking, further incubated with fluorescently labeled secondary antibodies/reagents (Alexa 594 and Alexa 647, Jackson Immunoresearch, West Grove, PA, USA; Neurotrace 500/525, ThermoFisher, Waltham, MA, USA; all at 1:500) at room temperature for two hours, and washed three times for 30 minutes in PBST. Tissues were mounted in Fluoromount-G (cat#0100-01, Southern Biotech, Birmingham, AL, USA) and coverslipped on microscope slides.

For each animal, 3–4 coronal tissue sections that included the AC, MGB, IC, CN, SOC and MNTB were imaged with a Leica Stellaris 5 Inverted Confocal Microscope (Leica Microsystems, Wetzlar, Germany) using a 10x objective lens as indicated in the figure legends. Imaging parameters were kept constant across all sections for each set of immunofluorescent labels. Experimenters were blinded to the genotypes and ages of the animals. All images were processed with ImageJ FIJI (NIH) and analyzed with a custom CellProfiler (Stirling et al., 2021) pipeline.

For quantification of total area covered by microglia or activated astrocytes, regions of interest (ROIs) outlining the above-mentioned structures were drawn on maximum z-projections of the acquired images and the corresponding masks were generated with a custom ImageJ plugin. Images were subsequently thresholded and binarized using the “Otsu” thresholding algorithm embedded in CellProfiler. The microglia and activated astrocytes were calculated as the ratio between the number of pixels thresholded over all pixels in the ROIs. In the present study, microglia coverage is presented as “% Microglia Coverage (Iba1)” and activated astrocyte coverage is presented as “GFAP Area Fraction (%)”.

For quantification of microglia activation, the CD68 images were thresholded and binarized in the same regions, and the overlap between CD68 and microglia was measured by the “RelatedObjects” function. The number of colocalized signal pixels was divided by the total microglia pixels to calculate the fraction of CD68-expressing microglia.

### Histology of the cochlea

Cochleae were dissected from euthanized mice at designated time points. The stapes was removed and a small incision was made at the apical tip of the cochlea for proper fluid exchange during immersion fixation with 4% paraformaldehyde (PFA) diluted in PBS. Tissues were transferred to decalcifying 0.1 M ethylenediaminetetraacetic acid (EDTA) at 4°C on a rocker for three days.

For cryosectioning, tissues were submerged in 30% sucrose/1x PBS overnight, embedded in optimal cutting temperature compound (OCT, Sakura Finetek USA, Torrance, California, USA), and frozen with liquid nitrogen. These tissues were sectioned at 20 μm with the 16 and 32 kHz regions visible and mounted onto slides (Fisherbrand Superfrost Plus, Thermo Fisher Scientific, Waltham, MA, USA). The 16 and 32 kHz regions were selected to analyze changes.

For immunohistochemical staining on cryosections, sections were washed with PBST at pH 7.4, and then blocked for 1 hour at room temperature in 5% normal donkey serum diluted in PBST. Antigen retrieval was performed prior to the immunostainings by boiling slides in 10 mM citric acid, pH 6, for 15 min at 20% microwave power. Antibody incubations were performed overnight at 4°C in blocking solution. The following primary antibodies were used in this analysis: mouse TUJ1 (anti-beta Tubulin 3, 1:100, R&D Systems, Minneapolis, MN, USA) and mouse anti- Calbindin 2 (Calb2, 1:100, MilliporeSigma, St. Lois, MO, USA). Sections were washed in PBST at room temperature prior to secondary antibody incubation overnight at 4°C in the dark. Alexafluor-conjugated secondary antibodies included 488, 594, and 647 (Thermo Fisher Scientific) and were diluted at 1:500 each. 4’6-diamidino- 2-phenylindole (DAPI) diluted at 1:10000 was added during the secondary antibody incubation. Sections were washed in PBST and mounted in Fluoromount-G (Southern Biotech) and coverslipped on microscope slides.

For the synapse analysis, whole mount preparation was preformed following cochlea dissection and decalcification. Cochleae were microdissected into three turns (apical, middle, and basal) as previously described (Montgomery and Cox, 2016). These pieces were frequency mapped using the ImageJ 64 (NIH) plug-in from Massachusetts Eye and Ear Infirmary, and immunostained for quantification. Antigen retrieval was performed by snap-freezing in liquid nitrogen, followed by thawing at room temperature for 1 hour. The following primary antibodies were used in this analysis: mouse anti-C-Terminal Binding Protein 2 (anti-CtBP2, 1:200, BD Biosciences, Franklin Lakes, NJ, USA), rabbit anti-Vesicular Acetylcholine Transporter (anti-VAT, 1:500, MilliporeSigma) and goat-anti Oncomodulin (anti-OCM, 1:500, Thermo Fisher Scientific).

Sections were imaged using an Olympus FV1000 (Olympus, Tokyo, Japan) laser scanning confocal microscope. For spiral ganglion neuron (SGN) analysis, images were acquired with 5 z-stacks which were centered at the sagittal plane of the section. The z-projection of the stacks were used for quantification. TUJ1-positive and Calb2-positive cells were counted by an experimenter blinded to animal information with the built-in cell counting function in ImageJ FIJI (NIH). The Type I SGN density was calculated by dividing the TUJ1-positive cell number by the area of the spiral ganglion region. Type I SGNs include three subtypes: Type Ia and Ib are Calb2 positive cells, while Type Ic is Calb2 negative cells (Shrestha et al., 2018). The Calb2-negative cell number was calculated by subtracting the TUJ1-positive cell number from the Calb2-positive cell number, then dividing by the TUJ1-positive cell number to calculate the proportion of Type 1c cells.

To assess the synapse loss in 5xFAD cochleae, the 8, 16, and 32 kHz regions from the whole mounts were imaged at 100x on the Olympus FV1000 microscope. The frequencies were selected given that significant hearing loss was present at those frequencies (Na et al., 2023). Images were converted (.oif to .ims) and imported into Imaris 9.3 Image Visualization & Analysis Software (Oxford Instruments, Abingdon, Oxfordshire, UK) for 3-D reconstruction. CtBP2 and VAT isosurfaces in inner cell regions and VAT isosurfaces in outer cell regions were thresholded and constructed. The number of CtBP2 isosurfaces and the volume of VAT isosurfaces were calculated with the Imaris built-in function. The inner hair cell nuclei were identified by the CtBP2 staining with the shape distinctive from ribbon synapses. The outer hair cells were identified with Oncomudulin staining.

### Behavioral assays

Mice were tested with a battery of behavioral assays in the following order: 1) open field testing (day 1); 2) novel object recognition (day 2); 3) fear conditioning (day 3). 14 days before behavioral testing, mice were switched to a reverse light/dark cycle room. For 3 days before behavioral testing, mice were transported from the colony room to the behavior room, handled for 5 minutes and then returned to the colony room within the same day. All experiments were carried out between 9 am to 5 pm. 5xFAD mice and their WT littermates within each cohort were tested on the same day for each experiment at each age. The same experimenter blinded to the genotypes of mice performed all the behavioral tests presented in this chapter. The ages of mice are as indicated in the figure legends.

#### Open field testing:

Open field testing was performed to measure anxiety and locomotor function. Each mouse was allowed to freely explore within a 31 × 31 cm box for 5 min. The periphery of the box was defined as 5 cm from the box edges and the center zone was defined as the remaining internal area. Distance (meters) and head entries into the center zone were measured automatically by ANY-Maze software (Stoelting, Wood Dale, IL, USA). Center zone entry was calculated by normalizing the total number of head entries into the center zone with the total distance traveled.

#### Novel object recognition:

Novel object recognition (NOR) was performed to assess short-term object recognition memory. During the habituation phase, each mouse was allowed to freely explore within a 31 × 31 cm box for 5 min containing two identical objects (ceramic doorknobs 5–6 cm in height and ~ 3 cm in width with white color) spaced ~ 15 cm apart. Objects and chambers were washed with 70% ethanol before each trial. Two hours after the habituation phase, each mouse was returned to the box containing the object to which it was previously exposed (familiar object) as well as a novel object (a plastic doorknob with black color and smaller shape). Placement of the novel object was randomized for each mouse. Mice were allowed to explore familiar and novel objects during a 5 min test that was videotaped for subsequent analysis using the ANY-Maze software. The scoring of NOR was based on the total time spent exploring the familiar object or the novel object, which was measured by ANY-maze software. The recognition index (RI) was calculated as (time with novel object/total interaction time) x 100.

#### Fear conditioning:

Cued and contextual fear conditioning was used to assess conditioned memory. On the conditioning day, each mouse was allowed to freely explore the conditioning chamber which consisted of a Plexiglass chamber and metal floor grid (Coulbourn Instruments, Whitehall, PA, USA). After 3 min, they were given 3 foot-shocks (0.5 mA for 2 sec each) during the presentation of white noise at 80 dB for 15 sec. 24 hours later, the mouse was placed back into the same chamber and its freezing responses was measured for 5 min to test its contextual long-term memory (familiar context session, referred to as “familiar”). 4 hours later, the mouse was placed in a novel context environment consisting of a 15 cm open-topped plastic cylinder with bedding on the floor for 3 min (novel context session, referred to as “novel”) followed by re-exposure to the white noise for 3 min, to test hippocampal-independent memory (tone test, referred to as “tone”). The freezing time in the first 30 sec of each session was divided by 30 and multiplied by 100 to calculate the freezing score in the familiar context, novel context, and tone test sessions. All data were video recorded and scored using ANY-Maze software.

### Machine learning classification

#### Feature extraction:

The ABRs evoked by 65 dB click stimuli were selected when all five waves were consistently present among all mice and this sound level was consistently above the hearing threshold for all mice tested. ABRs were recorded from 5xFAD mice and their WT littermates at 3M, 6M and 12M as well as APP/PS1 mice and their WT littermates at 13M. The ABRs from the 5xFAD mice at 12M, APP/PS1 mice at 13M and their littermates were grouped together and referred to as “12–13M” group in the machine learning classification and ABR waveform analysis. 18 variables were extracted from those ABRs and referred to as “features” in the machine learning classification, including Wave I amplitude (p1 amplitude), Wave I latency (p1 latency), Wave II amplitude (p2 amplitude), Wave II latency (p2 latency), Wave II to I amplitude ratio (p2:p1 ratio), Wave I to II latency interval (p2 – p1 interval), Wave III amplitude (p3 amplitude), Wave III latency (p3 latency), Wave III to I amplitude ratio (p3:p1 ratio), Wave I to III latency interval (p3 – p1 interval), Wave IV amplitude (p4 amplitude), Wave IV latency (p4 latency), Wave IV to I amplitude ratio (p4:p1 ratio), Wave I to IV latency interval (p4 – p1 interval), Wave V amplitude (p5 amplitude), Wave V latency (p5 latency), Wave V to I amplitude ratio (p5:p1 ratio), and Wave I to V latency interval (p5 – p1 interval).

#### Machine learning classification:

Machine learning classification and feature importance analysis were carried out with the CatBoost model (Prokhorenkova et al., 2018). Age groups were trained separately. In the training stage, the ABR data, including the data of all 18 variables mentioned above, and the genotypes of the mice from which the ABRs were recorded, were fed into the algorithm to train a model. Following the training, another set of ABR data, which does not include the genotypes, was then fit into the model, and the predicted/classified genotypes, as well as the probability of the prediction, were generated as the outcome. This step was repeated for all ABRs in the cross-validation step. The total outcome was then used to calculate the ROC curve, accuracy, sensitivity, and specificity.

Feature importance analysis calculates a score for the contribution of all the input features to the classification. In the present study, feature importance values were normalized so that the sum of importances of all features was equal to 100 (therefore the numbers were presented as a percentage). A higher score of feature importance indicated that this feature contributed more to the AD identification in our analysis, and therefore indicated a more pronounced difference between WT and AD mice in this variable.

#### Cross-validation:

Classification experiments were implemented using stratified 3-fold cross-validation to preserve the same percentage of samples for each class to improve robustness. In this step, the dataset was partitioned into 3 equal-sized subsamples (folds). A single subsample of the 3 folds was retained as the validation data for model testing; the remaining 2 subsamples were used as training data. Each subsample was classified by the machine learning model trained from a dataset not including this subsample itself, therefore a generalized estimation of the classification performance was achieved.

#### ROC and AUC:

The area under the receiver operating characteristic (ROC) curve (AUC) was calculated to evaluate the performance of classification on ABRs at different ages. The ROC curve was a plot of the true positive rate versus the false positive rate of a classification task. The different points on the curve corresponded to the different cutpoints of probability used to determine whether the genotype was AD or WT. A classification result that was better than a random classifier would reveal a ROC curve above the diagonal line on the ROC curve plot. The AUC, which was computed as the area under the ROC curve, is commonly used as an effective way to summarize the overall performance of the classification (Mandrekar, 2010). In general, an AUC of 0.5 suggested no discrimination (i.e., no ability to diagnose patients/subjects with and without the disease based on the test), 0.7 to 0.8 was considered acceptable, 0.8 to 0.9 was considered excellent, and > 0.9 was considered outstanding for a clinical diagnostic test (Mandrekar, 2010).

#### Accuracy, sensitivity and specificity:

Accuracy, sensitivity and specificity are commonly used to evaluate the performance of prediction and diagnosis. Accuracy was calculated by the total number of correctly classified subjects divided by the total number of subjects. The sensitivity was calculated by the total number of correctly classified positive subjects (5xFAD or APP/PS1 mice) divided by the total number of positive subjects. Specificity was calculated by the total number of correctly classified negative subjects (WT mice) divided by the total number of negative subjects.

The cross-validation, ROC plotting, and AUC calculation were performed with the built-in functions in the Python scikit-learn package (Buitinck et al., 2013).

### Statistical analysis

Sample sizes were calculated for sufficient power analysis. The sample size for each experiment is indicated within each figure legend. The researcher was blinded to the mouse genotypes and conditions for the ABR waveform analyses. The Shapiro Wilk test was used to assess normality for each data set, and nonparametric tests were used for all non-normal datasets. Unpaired student’s t-tests, the Mann-Whitney U rank sum test, a two-way ANOVA, and Kruskal-Wallis tests were used to compare differences across groups. Bonferroni adjustment and the Holm-Šídák multiple comparisons test were used for post-hoc analysis. Tests used for each experiment are indicated in the text. A p-value < 0.05 was considered statistically significant. Data are presented as modified box or bar plots (with error bars representing SEM). For all plots, individual data are presented as dots. In all figures, the p-values are defined as: no significance (ns), p ³ 0.05; * p < 0.05; ** p < 0.01, and *** p < 0.001 unless otherwise noted. Statistics and data plotting were performed in GraphPad Prism 9.3.1 and R 4.1.2.

## Results

### ABR abnormality identified AD in 5xFAD mice beginning at 6 months of age.

**Figure 1:**
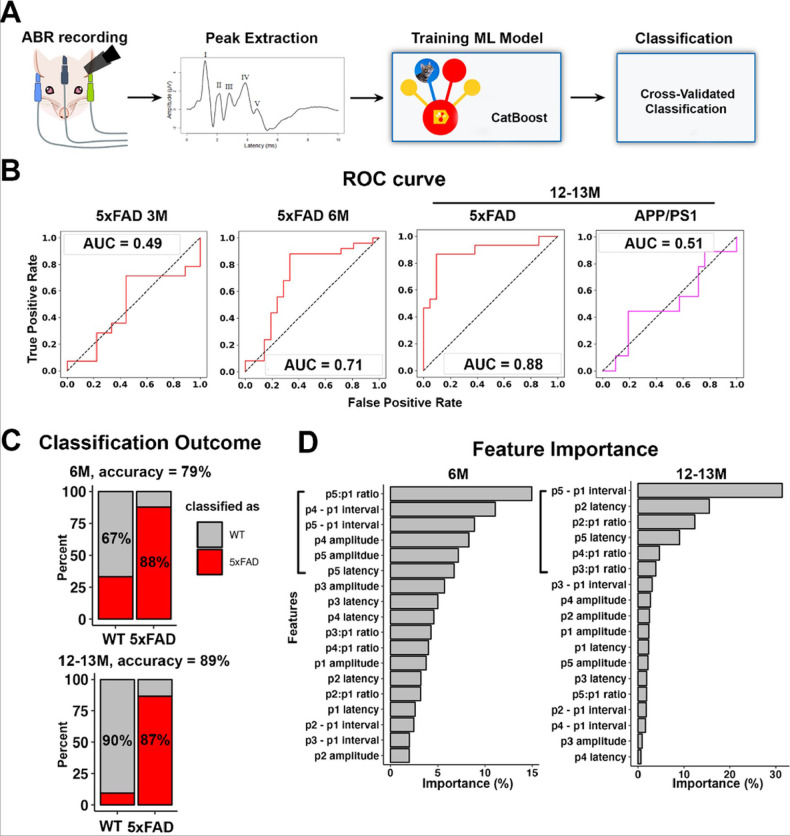
An ABR abnormality identified AD in 5xFAD mice beginning at 6M. A) The workflow of machine learning classification. B) ROC curves (in red for 5xFAD vs WT and magenta for APP/PS1 vs WT) for the classification of ABRs from mice at 3M (WT, n = 9; 5xFAD, n = 14), 6M (WT, n = 21; 5xFAD, n = 25), and 12–13M (WT, n = 21; 5xFAD, n = 15; APP/PS1, n = 9). C) Bar plots of the confusion matrix with the percentage of correctly classified objects in each genotype, i.e., the sensitivity and specificity of the classification, for WT and 5xFAD mice at 6M (top) and 12–13M (bottom). D) The feature importance maps of the classification for ABRs (left: 6M 5xFAD vs. WT; right: 12–13M 5xFAD vs. WT). The left square bracket highlights the top six features contributing to the classification of ABR into WT and 5xFAD genotypes.

We previously showed that the central auditory activity was disrupted in 5xFAD mice, a transgenic model with ectopic overexpression of amyloid-β, suggesting the application of central auditory activity change as an AD biomarker (Na et al., 2023). ABR waveform change analysis showed that 5xFAD mice showed significant changes in multiple variables (Figure S1–2), indicating that a combination of ABR variables could be assessed to diagnose AD. To evaluate whether AD could be diagnosed by ABR waveforms, and also to acquire a deeper understanding about the auditory brainstem activity changes induced by AD, machine learning classification was carried out on ABRs from mice at 3, 6, and 12–13M. Figure 1A shows the workflow for the classification: clicked-evoked ABRs were recorded from 5xFAD and APP/PS1 mice and their WT littermates at different ages as indicated in Figure 1B. The first five ABR waves were identified after recording, and their amplitude and latency were extracted. The ABR data were then fed into the CatBoost model, and ABRs were classified as WT or AD by the model after training as described later. Figure 1B shows the ROC curves for the cross-validated classification. For the classification with ABR data from 5xFAD mice and their WT littermates at 3M, the AUC of ROC curve was 0.49, suggesting that the classification is not better than a random classifier (an AUC score of 0.5 was expected from classification by chance alone). For the classification with ABR data from 5xFAD mice and their WT littermates at 6M, the AUC of ROC curve was 0.71 (Figure 1B), which is generally considered acceptable (Mandrekar, 2010). The classification yielded 79% accuracy (with 88% sensitivity and 67% specificity, Figure 1C top panel), which is comparable to the clinical diagnosis for AD (Beach et al., 2012). For the classification with ABR data from mice at 12–13M, the AUC of ROC curve further increased to 0.88 for the 5xFAD mouse prediction, with improved accuracy (89%), comparable sensitivity (87%), and improved specificity (90%) (Figure 1B, C bottom panel). These results suggest that the prediction performance improved along with disease progression. As with the APP/PS1 prediction, the AUC of ROC curve was 0.51 (Figure 1B), suggesting that the prediction was not reliable for this mouse strain.

Given that the machine learning classification revealed reliable results for 5xFAD mice at 6 and 12–13M, we further interpreted the classification by extracting the feature importance. For the classification on mice at 6M, we found that the top six features, which together took up more than 50% of the total importance (57%), were exclusively from Waves IV and V (Figure 1D, left). For the classification of mice at 12–13M, the top six features were from the waves following Wave I (Waves II, III, IV, and V; Figure 1D, right). While the latency or peak intervals reflect the conduction velocity of the pathway, the amplitude or amplitude ratio between Wave II – V to Wave I reflect the activity changes of the ABR neural generators (Eggermont, 2019). ABR Waves IV and V are suggested to be generated by regions including the IC and the SOC, while Waves II and III are proposed to be generated by the CN and SOC (Melcher and Kiang, 1996; Melcher et al., 1996; Land et al., 2016; Kim et al., 2022). Therefore, the feature importance map suggested that in 5xFAD mice, the activity change may occur in the higher levels of auditory pathway, such as the IC or SOC of 5xFAD mice earlier, then extend to the entire central auditory pathway along with the disease progression. We had previously showed that among ABR generators, the amyloid-β plaque was present in the IC of 5xFAD mice beginning at 6M (Na et al., 2023)., which is consistent with the feature importance map.

Multiple lines of evidence suggested that neuroinflammation might be the link between AD and the central auditory hyperactivity in 5xFAD mice. First, we previously performed a comprehensive examination on amyloid-b plaque deposition in the central auditory system of the 5xFAD mice, and found that amyloid-b plaque was prominent in the upper auditory brainstem of 5xFAD mice (Na et al., 2023). Neuroinflammation, which is typically characterized by microglia and astrocyte activation, has been suggested as the downstream effect of plaque deposition, therefore it is likely to occur along with the amyloid-b plaque observed in our 5xFAD mice (Long and Holtzman, 2019; Leng and Edison, 2020). Second, the pattern of plaque distribution is consistent with the activity change pattern suggested by the machine learning classification. Lastly, work by other groups have suggested that neuroinflammation is involved in mediating the excitation-to-inhibition (E/I) imbalance in the auditory system (Wang et al., 2019; Shulman et al., 2021), which is a potential cause for the central hyperactivity we observed in our 5xFAD mice (Na et al., 2023). Therefore, to further examine the correlation between AD and central auditory hyperactivity, we assessed the microglia and astrocyte activation in the central auditory pathway of AD mice.

### Microglia activation was prominent in the upper auditory brainstem of 5xFAD mice beginning at 6 months of age.

**Figure 2:**
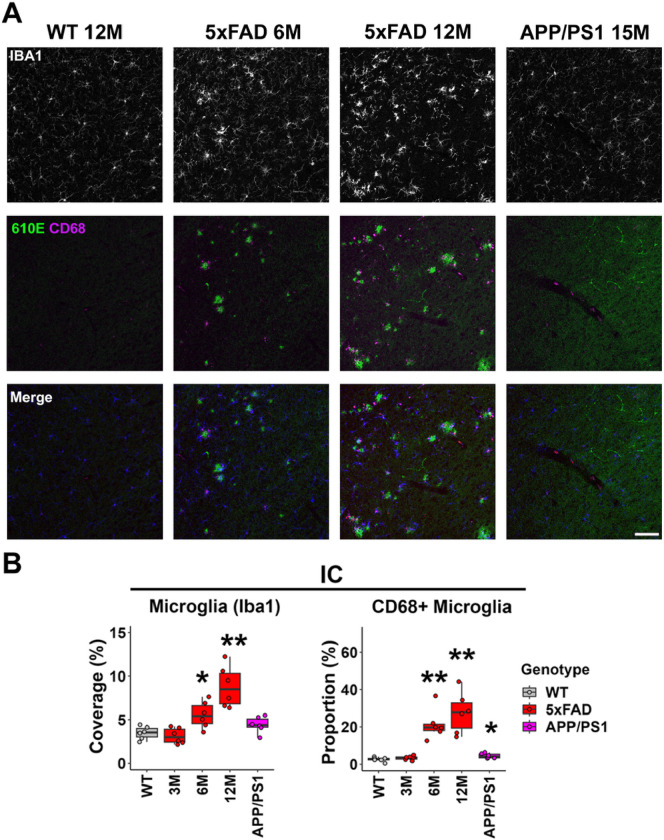
Microglia activation was prominent in the IC of 5xFAD mice beginning at 6M. A) Representative views of the IC from WT mice at 12M, 5xFAD mice at 3, 6, and 12M, and APP/PS1 mice at 13M. Top row shows IBA1 immunostaining (white), middle row shows amyloid-b immunostaining (610E, green) and CD68 immunostaining (magenta), and the bottom row shows the merged image, with IBA1 in blue. Scale bar = 100 μm. B) The percentage of area covered by microglia (left) and the proportion of CD68-positive microglia (right) are displayed (n = 6 for each group). Data are presented by modified box plots with jitter points to represent individual animals. Asterisks denote the significant differences between WT and AD mice: no significance (no asterisk), p ≥ 0.05; * p < 0.05; ** p < 0.01; and *** p < 0.001.

To examine the neuroinflammatory status in the central auditory system, we evaluated microglia activation using immunohistochemistry on brain sections. The microglia specific marker, Ionized calcium-binding adaptor molecule 1 (Iba1), was labelled to characterize the density of microglia in regions of interest. In the IC, the increase in microglia density became significant for 5xFAD mice starting at 6M (mean = 5.56%, p = 0.026, Wilcoxon test), and further increased by 12M (mean = 8.80%, p = 0.002, Wilcoxon test) (Figure 2A, top panel; B, left graph).

We previously observed differential central gain changes among 5xFAD mice and APP/PS1 mice at similar ages (Na et al., 2023). We proposed that the variable pattern of AD pathology may be key to this difference. In the present study, we examined the neuroinflammatory status in relevant regions of APP/PS1 mouse brains to test this hypothesis. The microglia density in the IC of APP/PS1 mice at 13M did not show a significant increase compared to WT mice (Figure 2A, top panel; B, left graph).

CD68 was labelled in brain sections to identify activated microglia (Hopperton et al., 2018). Sections were co-labeled with an antibody against amyloid b (610E) to reveal the location of plaques. The proportion of CD68-positive microglia showed a significant increase in the IC of 5xFAD mice at 6M (mean = 1.23%, p = 0.002, Wilcoxon test, Figure 2A middle panel; B right graph), consistent with our previous observation of amyloid-b plaque deposition in this region (Na et al., 2023). It further increased in these mice at 12M (mean = 2.46%, p = 0.002, Wilcoxon test, Figure 2A, middle panel; B, right graph). For both time points, CD68 immunoreactivity qualitatively associated with amyloid-b plaques (Figure 2A, center columns). For APP/PS1 mice, the increase of the CD68-positive microglia proportion was also significant in the IC; however, the level was much lower compared to 5xFAD mice at 6 and 12M (mean = 0.20%, p = 0.04, Wilcoxon test, Figure 2A, middle panel; B, right graph). Immunoreactivity for amyloid-b plaques were also reduced, as previously described (Na et al., 2023).

**Figure 3:**
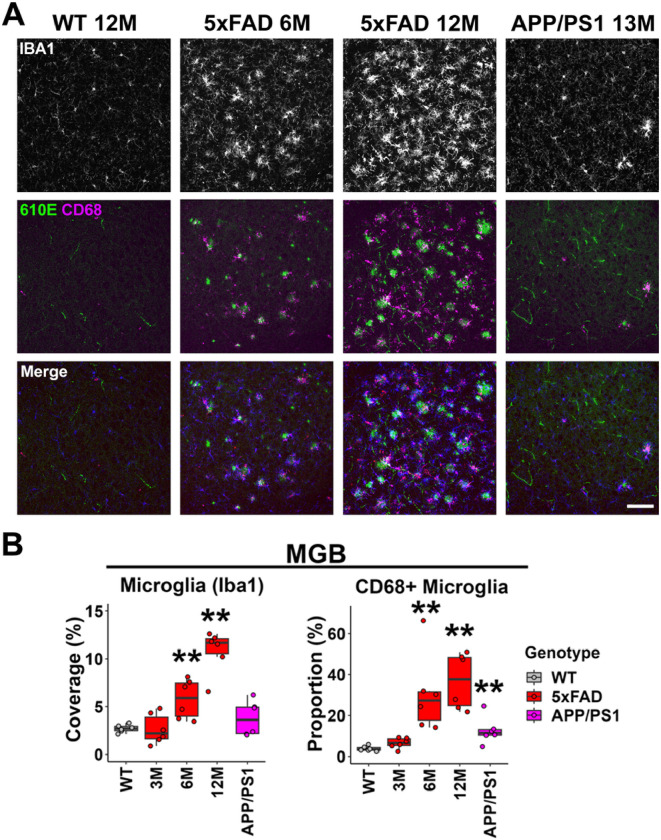
Microglia activation was prominent in the MGB of 5xFAD mice beginning at 6M. A) Representative views of the MGB from WT mice at 12M, 5xFAD mice at 3, 6, and 12M, and APP/PS1 mice at 13M. Top row shows IBA1 immunostaining (white), middle row shows amyloid-b immunostaining (610E, green) and CD68 immunostaining (magenta), and the bottom row shows the merged image, with IBA1 in blue. Scale bar = 100 μm. B) The percentage of area covered by microglia (left) and the proportion of CD68-positive microglia (right) are displayed (n = 6 for each group). Data are presented by modified box plots with jitter points representing individual animals. Asterisks denote the significant differences between WT and AD mice: no significance (no asterisk), p ≥ 0.05; * p < 0.05; ** p < 0.01; and *** p < 0.001.

The microglia density (Iba1) and proportion of CD68-positive microglia also showed a significant increase in the MGB of 5xFAD mice beginning at 6M (microglia coverage: 6M 5xFAD, p = 0.002; 12M 5xFAD, p = 0.002; Wilcoxon test; CD68+ microglia proportion: 6M 5xFAD, p = 0.002; 12M 5xFAD, p = 0.002; Wilcoxon test, Figure 3). CD68+ cells qualitatively associated with amyloid-b plaques (Figure 3A, central row). In the MGB of APP/PS1 mice, similar to the IC, the microglia density increase was not significant (Figure 3A top panel; B, left graph). The CD68+ microglia proportion showed a significant increase in this genotype (Figure 3A middle panel; B right graph), even though significant levels amyloid-β plaque was not detected in our previous assay (Na et al., 2023). With the higher resolution images presented here, it is clear that the CD68+ cells are associating with the low levels of plaque present (Figure 3).

Given that both the density and CD68 expression of microglia showed significant increases in the IC and MGB of 5xFAD mice, we conclude that microglia activation was significant in the upper auditory brainstem of 5xFAD mice beginning at 6M. The p-values for all comparisons can be found in Tables S3-S4.

### Microglia activation was prominent in the AC of both of 5xFAD and APP/PS1 mice.

**Figure 4:**
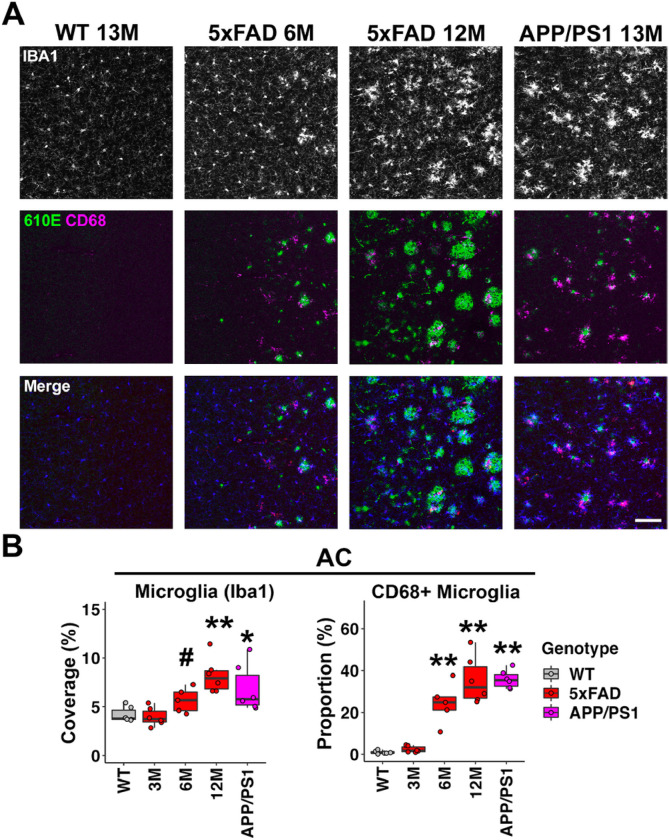
Microglia activation was prominent in the AC of both of 5xFAD and APP/PS1 mice. A) Representative views of the AC from WT mice at 12M, 5xFAD mice at 3, 6, and 12M, and APP/PS1 mice at 13M. Top row shows IBA1 immunostaining (white), middle row shows amyloid-b immunostaining (610E, green) and CD68 immunostaining (magenta), and the bottom row shows the merged image, with IBA1 in blue. Scale bar = 100 μm. B) The percentage of area covered by microglia (left) and the proportion of CD68-positive microglia (right) are displayed (n = 6 for each group). Data are presented by modified box plots with jitter points representing individual animals. Asterisks denote the significant differences between WT and AD mice: no significance (no asterisk), p ≥ 0.05; * p < 0.05; ** p < 0.01; *** p < 0.001 and # p = 0.052.

Although not proposed as an ABR generator, the AC potentially regulates the activity of multiple levels of the auditory brainstem via corticofugal projections (Terreros and Delano, 2015). We therefore examined microglia activation in the AC of AD mice. For 5xFAD mice at 6M, the microglia density showed an increasing trend, but the difference was not significant (mean = 5.67%, p = 0.052, Wilcoxon test, Figure 4A, B, left graph); it further increased in mice at 12M and became significant (mean = 8.22%, p = 0.002, Wilcoxon test, Figure 4A, B left graph). The increase in microglia density was also significant in the AC of APP/PS1 mice at 13M (p = 0.015, Wilcoxon test, Figure 4A, B, left graph).

The increase of microglial phagocytic activity was evident in the AC of 5xFAD mice at 6 and 12M (6M: p = 0.004, 12M: p = 0.002, Wilcoxon test, Figure 4A, B right graph). Microglia in the AC of APP/PS1 also showed a significant increase in phagocytosis (p = 0.002, Wilcoxon test, Figure 4A, B, right graph), which is consistent with the previous observation of plaque deposition in this region of APP/PS1 mice. CD68 immunoreactivity in the AC also associated with amyloid-b plaques (Figure 4A, center row).

Taken together, those analyses show that microglia activation was evident in the AC of both of 5xFAD and APP/PS1 mice. The p-values for all comparisons can be found in Table S3-S4. Given that central gain increase was not detected in APP/PS1 mice, the correlation between neuroinflammation in the AC and the central gain increase in auditory brainstem appears to be minimal.

### Astrocyte activation was prominent in the upper auditory brainstem of 5xFAD mice beginning at 6 months of age.

**Figure 5:**
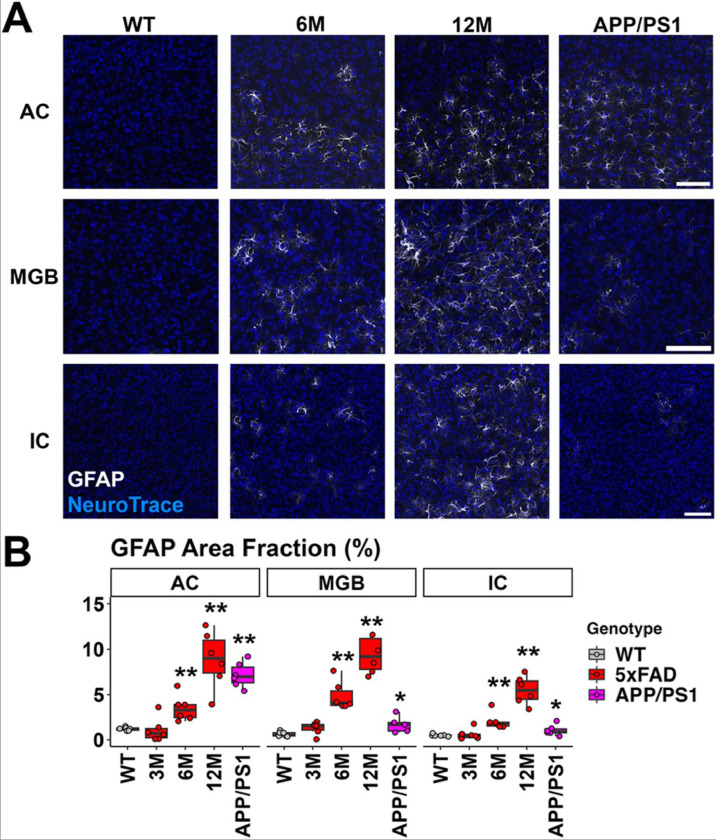
Astrocyte activation was prominent in 5xFAD mice beginning at 6M in the auditory cortex and upper auditory brainstem. A) Representative views of the AC, MGB and IC from WT mice at 12M, 5xFAD mice at 3, 6, and 12M, and APP/PS1 mice at 13M. Scale bar = 100 μm. B) The percentage of area covered by GFAP is displayed (n = 6 for each group). Data are presented by modified box plots with jitter points representing individual animals. Asterisks denote the significant differences between WT and AD mice: no significance (no asterisk), p ≥ 0.05; * p < 0.05; ** p < 0.01; and *** p < 0.001.

Astrocyte reactivity is another hallmark of AD and part of the neuroinflammation process. A well-established marker for reactive astrocytes is the upregulation of glial fibrillary acidic protein (GFAP) (Leng and Edison, 2020). Therefore, we labelled GFAP in brain sections to characterize the astrogliosis in the central auditory pathway of AD mice. Astrocyte activation is evident in the AC of 5xFAD mice and APP/PS1 mice (Figure 5A top panel). As with the subcortical regions such as the MGB and IC, astrocyte activation appeared to be more prominent in the 5xFAD mice, compared to the APP/PS1 mice (Figure 5A, middle and bottom panels). The area covered by the GFAP signal was quantified and is presented in Figure 5B. In the AC, the increase of GFAP coverage was significant in 5xFAD and APP/PS1 mice compared to the WT controls (6M 5xFAD: p = 0.002, 12M 5xFAD: p = 0.002, APP/PS1: p = 0.002, Wilcoxon test). In the MGB, the increase of GFAP coverage was also significant in 5xFAD and APP/PS1 mice (6M 5xFAD: p = 0.002, 12M 5xFAD: p = 0.002, APP/PS1: p = 0.017, Wilcoxon test). However, the mean value was much lower in the APP/PS1 mice (6M 5xFAD, mean = 4.84%; 12M 5xFAD, mean = 11.03%; APP/PS1, mean = 1.97%). In the IC, 5xFAD mice showed an increase of GFAP expression beginning at 6M (compared to WT, 6M 5xFAD: p = 0.002, 12M 5xFAD: p = 0.002). APP/PS1 mice showed a GFAP expression increase in the IC, but at lower level compared to 5xFAD mice (APP/PS1, p = 0.04, mean = 1.06%; 6M 5xFAD, mean = 2.07%; 12M 5xFAD, mean = 5.47%; Wilcoxon test). The p values for all comparisons can be found in Table S5. Overall, we conclude that astrocyte activation parallels the microglia activation in the auditory pathway of AD mouse models: it was significant in the IC, MGB, and AC of 5xFAD mice beginning at 6M, and only in the AC of APP/PS1 at 13M.

### 5xFAD mice showed increased level of microglia coverage but not activation in the SOC and MNTB at 12 months of age.

**Figure 6:**
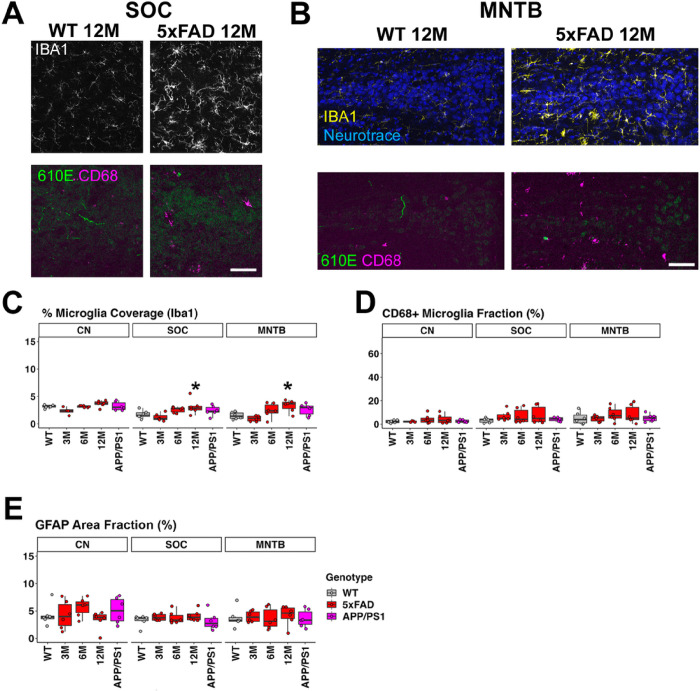
5xFAD mice showed increased microglia coverage but not microglial activation in the SOC and MNTB at 12M. A) Representative views of the SOC in 5xFAD and WT mice at 12M, with labels for Iba1 (white, top), amyloid-b(green, 610E, bottom) and CD68 (magenta, bottom). B) The MTNB (triangular shape of a neuron cluster) in 5xFAD and WT mice at 12M, with labels for NeuroTrace (neurons, blue, top), Iba1 (yellow, top), amyloid-b (green, 610E, bottom) and CD68 (magenta, bottom). The microglia coverage quantification (C), proportion of CD68-positive microglia (D), and activated astrocyte coverage (E, GFAP Area Fraction) in the CN, SOC, and MNTB are also presented (n = 6 for each group). The genotypes for (C-E) are indicated in the legend of (E). Scale bar = 100 μm. Asterisks denote the significant differences between WT and AD mice: no significance (no asterisk), p ≥ 0.05; * p < 0.05; ** p < 0.01; and *** p < 0.001.

Given the possible functionality change at lower levels of the auditory brainstem in 5xFAD mice at 12M, we examined microglia and astrocyte activation in the SOC, MNTB, and CN. We found that 5xFAD mice at 6 and 12M, and APP/PS1 mice showed a higher mean of microglia coverage in the SOC and MNTB compared to WT mice (SOC: mean = 1.713% for WT 12M, mean = 2.568% for 5xFAD 6M, mean = 3.094% for 5xFAD 12M, and mean = 2.413% for APP/PS1 13M; MNTB: mean = 1.474% for WT 12M, mean = 2.445% for 5xFAD 6M, mean = 3.264% for 5xFAD 12M, and mean = 2.604% for APP/PS1 13M, Figure 6C). The change is significant in 5xFAD mice at 12 months of age (Figure 6 A–C; p = 0.041 for SOC; p = 0.015 for MNTB). However, the proportion of CD68-positive microglia did not show a significant increase in the SOC or MNTB of any AD mice (Figure 6D). No significant levels of amyloid plaque were detected (Figure 6A, B), consistent with previous assay (Na et al., 2023). Astrocyte activation was also not significant in the SOC or MNTB of any AD mice (Figure 6E). In the CN, the percentage of microglia coverage, proportion of CD68-positive microglia, and astrocyte activation were not significant in any AD mice (Figure 6C–D). The p-values for all comparisons can be found in Table 3.S3 – S5. Overall, our observations suggest increased microglia coverage in the SOC and MNTB of 5xFAD mice at 12M, but further analysis did not support an interpretation of glial activation.

### No correlation observed between changes of peripheral neuronal structures and central auditory hyperactivity.

**Figure 7:**
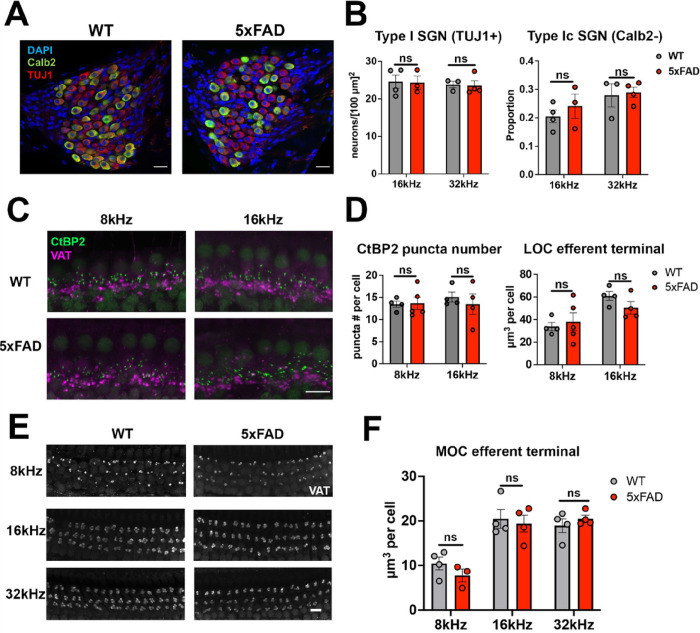
No correlation observed between changes of peripheral neuronal structures and central auditory hyperactivity. A) Representative view of SGN labelling in 5xFAD and WT mice at 12M. Images show the 16 kHz regions, with labels for DAPI (blue), Calb2 (green), and TUJ1 (red). B) Quantification of Type I SGN density (left) and the proportion of Type Ic SGNs (right). C) Representative view of afferent and efferent terminal labelling in cochlear whole mounts from 5xFAD and WT mice at 12M, with labels for CtBP2 (green) and VAT (magenta). Tonotopic cochlear regions for 8 kHz (left column) and 16 kHz (right column) are shown. D) Quantification of CtBP2 puncta number per inner hair cell (left) and mean VAT terminal volume per inner hair cell (right). E) Representative view of efferent terminal labelling (VAT in white color) in cochlear whole mounts from 5xFAD and WT mice at 12M. The 8 (top row), 16 (middle row), and 32 (bottom row) kHz regions are shown. F) Quantification of VAT terminal volume (μm^3^) per outer hair cell (OHC). Asterisks denote the significance of differences between WT and 5xFAD mice: no significance (ns), p ≥ 0.05; * p < 0.05; ** p < 0.01; and *** p < 0.001, Student’s t-test or Wilcoxon test. Scale bar = 10 μm for(A), (C) and (E).

We previously observed an ABR Wave I amplitude reduction in 5xFAD mice at 12M (Na et al., 2023), which is generally considered a reflection of synapse loss or SGN degeneration in the cochlea (Kohrman et al., 2020). SGN and synapse loss are also a possible cause for central auditory hyperactivity (Auerbach et al., 2014; Schrode et al., 2018). We therefore examined the synapse loss and SGN degeneration in the cochleae of 5xFAD mice and their WT littermates at 12M (Figure 7). TUJ1, the type I SGN-specific marker (Sekerková et al., 2008), was labelled in the spiral ganglions of cochleae. These neurons were co-labelled with Calb2, a marker differentially expressed in subtypes of type I SGNs (Figure 7A) (Shrestha et al., 2018). Type Ic SGNs, which are characterized as Calb2-negative neurons, are more vulnerable to insults and possibly the main source of the Wave I amplitude (Furman et al., 2013; Shrestha et al., 2018). The 16kHz region was a primary focus, since it showed the greatest change at the onset of hearing loss (9M) in 5xFAD mice (Na et al., 2023). The 32kHz region showed significant hearing loss in 5xFAD mice at 12M (Na et al., 2023), and therefore it was included in this assay. 5xFAD mice did not show significant changes in either region (Type I SGN density: 16 kHz, p = 0.93; 32 kHz, p = 0.86; Type Ic proportion: 16 kHz, p = 0.45; 32 kHz, p > 0.99; Unpaired Student’s t-tests and Wilcoxon tests) (Figure 7B). The 8kHz region is another region which showed significant change at the onset of hearing loss in 5xFAD mice (Na et al., 2023). However, it was not included in the same experiment set due to the technical difficulty.

Synapse loss, which is considered an event prior to SGN loss (Kujawa and Liberman, 2009), was further examined in those mice. CtBP2, the presynaptic ribbon-marker, was labelled in whole mount cochleae to characterize afferent synapse loss (Figure 7C). The CtBP2 number of puncta per inner hair cell was quantified (Figure 7D). The 8 and 16kHz regions were a main focus of analysis given that those regions showed significant difference at the onset of hearing loss in 5xFAD mice. Synapse loss was not significant in either region in those mice (8 kHz, p = 1; 16 kHz, p = 0.89; Wilcoxon tests).

Cholinergic lateral olivocochlear (LOC) efferents, which innervate the auditory nerve dendrites beneath the inner hair cells (IHCs), regulate auditory nerve activity and potentially protect the auditory nerve (Wu et al., 2020; Lauer et al., 2022). Therefore, we also examined the LOC efferent terminals by labelling with VAT (Figure 7C). Individual terminals were indistinguishable with the current resolution, and therefore the average VAT-positive terminal volume was quantified (Figure 7D, right graph). 5xFAD mice did not show a significant loss of LOC efferents in 8 and 16 kHz regions (8 kHz, p = 0.72; 16 kHz, p = 0.43; Unpaired Student’s t-tests).

The medial olivocochlear (MOC) efferents, which innervate the outer hair cells (OHCs), protect hair cells from aging and noise damage (Maison et al., 2013; Liberman et al., 2014). The MOC efferents have also been proposed to contribute to hearing in noise (Lauer et al., 2022), in which the 5xFAD mice showed significant amyloid accumulation (Na et al., 2023). Therefore, we examined the MOC efferent terminals which were labelled by VAT in outer hair cell regions (Figure 7E). The quantification of the average VAT terminal volume per hair cell is shown in Figure 7F. 5xFAD mice did not show a significant change in either 8, 16, or 32 kHz regions (8 kHz, p = 0.25; 16 kHz, p = 0.71; 32 kHz, p = 0.40; Unpaired Student’s t-tests).

Overall, the SGN, synapse, and efferent losses were not significant in 5xFAD mice. We conclude that no correlation was observed between peripheral neuronal changes and auditory dysfunctions in 5xFAD mice, including central auditory hyperactivity, hearing loss and hearing in noise deficit.

### Central auditory gain increase coincided with behavioral changes in 5xFAD mice.

The AD-induced central auditory gain increase suggested its application as a biomarker for AD diagnosis. However, whether it precedes the behavioral deficit was unclear. In the present study, we performed a battery of behavioral assays, including fearing conditioning, the open field test, and NOR on 5xFAD mice at 3, 6, and 12M, to examine this question.

The cued fear conditioning test is commonly used to evaluate the hippocampal-independent memory (Curzon et al., 2009). The freezing score in the novel context and tone test sessions were examined to evaluate each mouse’s performance following cued fear conditioning. The session difference between novel context and tone test sessions were significant for mice at 3, 6, and 12M, suggesting that the training was successful (3, 6, and 12M, p < 0.001; two-way ANOVA; Figure 3.8A). 5xFAD mice performed worse in the tone test at 6 and 12M compared to their WT littermates (3M, p = 0.39; 6M, p = 0.0002; 12M, p = 0.0016; two-way ANOVA followed by Bonferroni post-hoc analysis; Figure 3.8A, middle and right graphs). The hearing loss and ABR Wave I amplitude reduction were not significant in 5xFAD mice at 6M (Na et al., 2023), suggest that this phenotype is independent from peripheral auditory dysfunction at least at this age.

**Figure 8:**
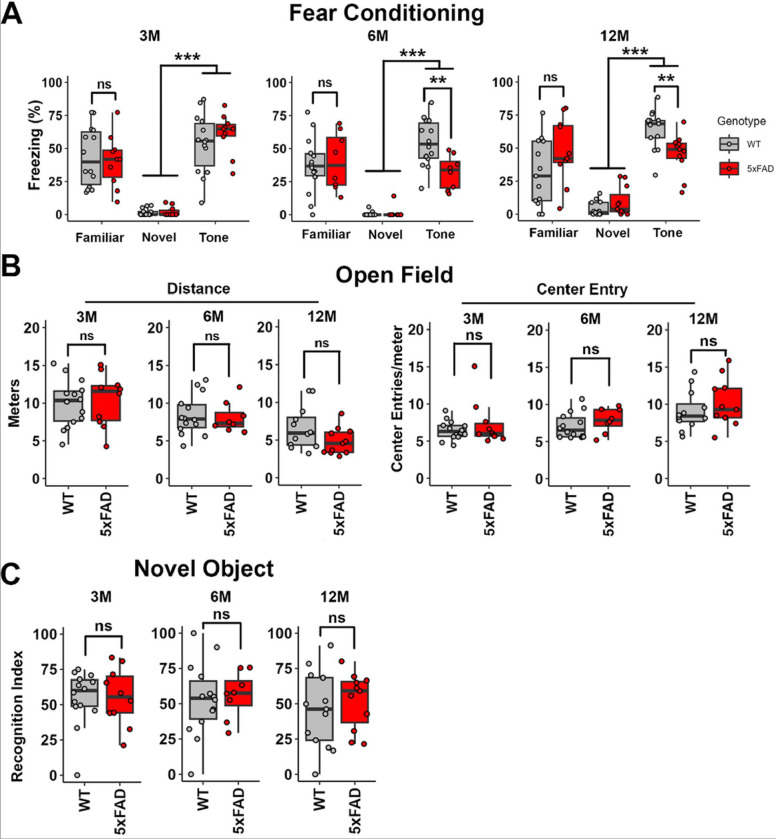
Central auditory gain increase coincided with behavioral changes in 5xFAD mice. A) Quantification of cued and contextual fear conditioning test performance in 5xFAD mice and their WT littermates at 3, 6, and 12M (3M: WT, n = 14; 5xFAD, n = 10. 6M: WT, n = 14; 5xFAD, n = 8. 12M: WT, n = 12, 5xFAD, n = 11). Scores in the familiar context session (Familiar), novel context session (Novel), and tone test session (Tone) are shown. B) Quantification of open field test performance, with total travelling distance (Distance, left panel) and tendency to explore the center zone (Center Entry, right panel) scored. C) Quantification in the NOR test. Asterisks denote the significance of differences between WT and 5xFAD mice: no significance (ns), p ≥0.05; * p < 0.05; ** p < 0.01; and *** p < 0.001, Student’s t-test, Wilcoxon test, or two-way ANOVA.

The freezing score in the familiar context session was examined to evaluate contextual long-term memory in 5xFAD mice at 3, 6, and 12M (Figure 8A), which is considered as a hippocampal-dependent function (Curzon et al., 2009). 5xFAD mice did not show a significantly worse performance at any stage compared to their WT littermates (3M, p = 0.69; 6M, p = 0.76; 12M, p = 0.11; Wilcoxon test).

The open field testing was performed to evaluate the locomotor activity and anxiety levels of 5xFAD mice. Locomotor activity was evaluated by scoring the total distance travelled during the test session (Figure 8B, left graphs). 5xFAD mice did not perform significantly worse than WT littermates at any stage (3M, p = 0.76; 6M, p = 0.82; 12M, p = 0.079; Unpaired Student’s t-test and Wilcoxon test). The center entry was scored to evaluate the tendency of exploring the center zone. A reduced anxiety level has been correlated to an increased tendency to explore the center zone (Kraeuter et al., 2019). 5xFAD mice did not show significant changes in center entry at any stage compared to WT littermates (3M, p = 0.93; 6M, p = 0.30; 12M, p = 0.41; Student’s t-test or Wilcoxon test; Figure 8B, right graphs).

We used the NOR test to evaluate the recognition memory (Antunes and Biala, 2012) of 5xFAD mice. The recognition index was scored for mice at 3, 6, and 12M (Figure 8C). 5xFAD mice did not perform significantly worse at any stage compared to their WT littermates (3M, p = 0.89; 6M, p = 0.66; 12M, p = 0.47; unpaired Student’s t-test and Wilcoxon test).

Overall, we detected significant behavioral change in 5xFAD mice beginning at 6M in the cued fear conditioning test. The central auditory gain increase temporally correlates with this change (Na et al., 2023).

## Discussion

In the present study, we evaluated whether an ABR abnormality can identify AD in the transgenic mouse models 5xFAD and APP/PS1 with machine learning classification. The classification produced 79% accuracy in 5xFAD mice at 6M and 89% accuracy at 12M. Further interpretation of this classification suggests that the activity change was pronounced in the upper auditory brainstem of 5xFAD mice at 6M. Histological examination revealed significant microglia and astrocyte activation in nuclei of the upper auditory brainstem, including the IC and MGB. In addition, no correlation was observed between changes in peripheral structures and central auditory hyperactivity. Taken together, this evidence further supports the hypothesis that the presence of AD hallmarks in brainstem induces central auditory hyperactivity. We speculate that AD-driven changes to inhibitory signaling in the auditory brainstem disrupt auditory processing, which relies on precisely coordinated temporal firing.

### Neuroinflammation as a link between central auditory hyperactivity and AD

In the present study, we characterized microglia and astrocyte activation in the auditory brainstem. It is unsurprising that neuroinflammation was present in the IC and MGB, given that we documented significant amyloid-b plaque deposition in those regions (Na et al., 2023). However, confirming that amyloid-b plaque deposition in those regions is sufficient to induce reactive gliosis is important for understanding the central auditory hyperactivity in 5xFAD mice. The imbalance of excitatory and inhibitory signaling has been characterized as the cause of central auditory hyperactivity (Auerbach et al., 2014). The upregulation of pro-inflammatory factor, TNF-α, is currently the only known factor able to mediate this imbalance in the auditory system (Wang et al., 2019; Shulman et al., 2021). It is likely to happen in the regions where microglia activation is triggered by amyloid-b plaque deposition (Leng and Edison, 2020). Further investigation is necessary to better understand this correlation. Moreover, assessing inflammation and AD hallmarks in MCI and early AD patients will be key to relating these findings to human health.

### AD-related hearing loss in patients and animal models

Few reports exist on deafness caused by lesion in the auditory brainstem in humans (Egan et al., 1996; Hoistad and Hain, 2003; Musiek et al., 2004). However, a recent study showed that among subjects with AD, individuals that reported hearing loss had smaller brainstem volumes than individuals without hearing loss, suggesting that brainstem atrophy might be the link between hearing loss and AD (Llano et al., 2021). Studies in animal models revealed a mechanism by which central hearing loss is possible: lesions in the SOC that led to a complete loss of LOC and MOC efferents can accelerate age-related hearing loss, and increase the susceptibility of the cochlea to noise exposure (Maison et al., 2013; Liberman et al., 2014). While the SOC is not directly impacted by AD hallmarks in this study or in human patients (Ohm and Braak, 1989; Na et al., 2023), disruption in regulation from higher auditory pathway levels by AD pathology is a possibility (Terreros and Delano, 2015).

### Behavioral changes in 5xFAD mice: Discussion of consistency and inconsistency

To examine whether an ABR abnormality occurs before behavioral deficit, we performed a series of behavioral assays to evaluate multiple aspects of behavioral changes in 5xFAD mice at different ages. 5xFAD mice have been reported to develop a number of age-related behavioral phenotypes within the time course of our observation (3–12M), including memory impairment (4–5M), reduced emotionality/anxiety (6M), motor deficits (9M), and recognition impairment (10M) (Oakley et al., 2006; Jawhar et al., 2012; Locci et al., 2021).

In the present study, memory impairment was evaluated by a fear conditioning test (Figure 8). Previous studies reported that 5xFAD mice showed significantly worse performance in a contextual fear conditioning test beginning at 6M (Ohno, 2009). However, this test did not reveal significant differences between 5xFAD mice and their WT littermates from 3 to 12M (Figure 8A) in our assay. Similarly, a more recent study, which performed comprehensive phenotyping of 5xFAD mice (Forner et al., 2021), showed that the performance change was insignificant in 5xFAD mice at 4, 8, and 12M. Our results are more consistent with the more recent study.

The cued fear conditioning test, which is commonly used to evaluate the hippocampus independent memory, was also carried out in the present study. 5xFAD mice performed worse at 6 and 12M (Figure 8), which is consistent with previous studies (Bouter et al., 2014; Yang et al., 2017). The difference in freezing scores between the novel session and tone test session demonstrated that 5xFAD mice were successfully trained (Figure 8). The insignificant change of ABR threshold in 5xFAD mice at 6M (Na et al., 2023) suggests that impaired performance of those mice in this test was not due to the inability to detect the auditory cue. However, whether this deficit was due to amygdala dysfunction should be interpreted carefully. The input from the AC and MGB to the amygdala is considered the major contributor to the formation of memory related to both safe and threatening cues (Ferrara et al., 2017). The functions of the AC and MGB in 5xFAD mice were potentially impaired due to the amyloid-b plaque deposition and microglia activation (Na et al., 2023) and Figures 3 and 4). Therefore, the worse performance of 5xFAD mice in the cued fear conditioning test could be due to reduced input instead of amygdala dysfunction. Nevertheless, the behavioral change of 5xFAD mice was significant in this test.

5xFAD mice were reported to show reduced anxiety starting at 6M (Jawhar et al., 2012) in the elevated-plus maze test. When anxiety was measured using the open field test, results varied. In some studies, 5xFAD mice spent more time in the center zone at 7M (Belaya et al., 2020) or 8M (Forner et al., 2021), but not at 12M or later (Forner et al., 2021), suggesting reduced anxiety in those mice. Other studies showed that the 5xFAD mice spent less time in the center, or showed less tendency to explore the center at 10 or 11–13M (Locci et al., 2021; O’Leary and Brown, 2022). We saw no change in anxiety levels in 5xFAD mice at 3, 6, or 12M (Figure 8). Similarly, we performed the NOR test to evaluate the recognition memory of 5xFAD mice. A previous study showed that worse performance of 5xFAD mice was expected at 10M (Locci et al., 2021). However, in our assay, 5xFAD mice showed no significant performance change at 3, 6, or 12M (Figure 8).

Collectively, with this battery of behavioral assays, we observed significant behavioral change in 5xFAD mice only in the cued fear conditioning test. Although behavioral changes were expected in open field and NOR tests, we found no effect of genotype. A review of existing literature revealed inconsistency of animals’ performance in those tests among different studies. This suggests that despite the best efforts to standardize the procedure, the outcome of these behavioral assays can vary between different experimental environments and even litters of mice. In addition, the 5xFAD mice used in many studies possessed either a C57BL/6J congenic background or a B6SJLF1/J hybrid background. To the best of our knowledge, the genetic background used in our study, the CBA/B6 (CBA and C57BL/6J hybrid background), is a novel strain for these assays. Furthermore, slight differences in experiment protocol may also contribute to inconsistency. For example, in the NOR protocol applied by (Locci et al., 2021), habituation was carried out for three days, while in our protocol, we only used one day. This could contribute to unrevealed differences between our behavioral assays, although the pathological changes in CNS were obvious (Na et al., 2023).

Overall, we conclude that the behavioral changes of 5xFAD mice observed in the present study are consistent with previous studies and independent of hearing loss. However, whether the central auditory hyperactivity in 5xFAD mice precedes cognitive deficits related to hippocampus or amygdala dysfunction requires further investigation.

### ABR abnormality as a predictor for AD in combination with machine learning

Advances in artificial intelligence are growing at a remarkable rate due to the rapid development of machine learning algorithms. The power of machine learning algorithms lies in their ability to capture subtle changes and handle high-dimensional data (i.e., the dataset with the number of features/variables close to or larger than the number of observations). Machine learning algorithms have been widely applied to disease diagnostics in combination with approaches yielding results with complex features, such as imaging or Raman spectroscopy (Latif et al., 2019; Qi et al., 2023). In the present study, combined with a machine learning algorithm, an ABR abnormality was able to diagnose AD in 5xFAD mice when AD hallmarks were distributed to the auditory brainstem. It should be noted that the diagnosis was carried out using the ABR recordings evoked by click at only one sound level (65dB), which can be acquired in a timely manner (less than 1 min in mouse). This evidence suggests that ABR is a low-cost, non-invasive, and rapid AD biomarker when combined with machine learning.

The interpretability of some machine learning algorithms can facilitate the identification of changing features, which has been applied in explaining diagnostic results such as Raman spectrum from AD brain tissues (Wang et al., 2022). In the present study, the machine learning algorithm identified the salient features of ABR that were changing in 5xFAD mice, which were further confirmed by histological analysis. This result suggests that the application of this combined approach can learn the functionality changes of the auditory brainstem in AD patients at different stages or with differential AD hallmark distributions. In addition, advanced approaches such as parallel ABR can perform recordings in humans at multiple frequencies at a faster rate (speedup ratio of 6.0 comparing to conventional ABR) (Polonenko and Maddox, 2019). In the present study, validation of diagnostic performance was only limited to click-evoked ABR and in mouse models. Those kinds of advanced approaches will allow us to rapidly obtain more comprehensive information about ABR changes in human. Thus, by combining advanced approaches and machine learning, serial assessment of ABR could help us acquire new insights into neuropathology changes in AD and assist diagnosis. That information can also provide a possible window for the treatment of AD-related CAPD, thereby improving the quality of life for AD patients.

## Conclusion

Overall, we conclude that neuroinflammation, which is proposed as the link between AD and central auditory hyperactivity in 5xFAD mice, temporally and spatially correlates with the central auditory hyperactivity in those mice. The reliable performance of AD identification with a combination of ABRs and machine learning algorithm suggests the application of this approach in AD diagnosis. Moreover, further investigation into AD-driven pathology in the thalamus and brainstem in human patients might reveal if these structures are less robust to damage than the cortex.
